# Chemerin Regulates Epithelial Barrier Function of Mammary Glands in Dairy Cows

**DOI:** 10.3390/ani11113194

**Published:** 2021-11-09

**Authors:** Yutaka Suzuki, Sachi Chiba, Koki Nishihara, Keiichi Nakajima, Akihiko Hagino, Won-Seob Kim, Hong-Gu Lee, Tomonori Nochi, Toru Suzuki, Sang-Gun Roh

**Affiliations:** 1 Research Faculty of Agriculture, Hokkaido University, Kita-9 Nishi-9, Kita-ku, Sapporo 060-8589, Japan; yutasuzu@anim.agr.hokudai.ac.jp; 2 Graduate School of Agricultural Science, Tohoku University, 468-1 Aramaki Aza Aoba, Aoba-ku, Sendai 980-0842, Japan; oun38c4d2.x4869t@gmail.com (S.C.); kokokokoki197@gmail.com (K.N.); akihiko.hagino.b8@tohoku.ac.jp (A.H.); nochi@tohoku.ac.jp (T.N.); suzukitr42195@gmail.com (T.S.); 3 NARO Hokkaido Agricultural Research Center, Hitsujigaoka 1, Toyohira-ku, Sapporo 062-8555, Japan; k1nakaji@yahoo.co.jp; 4 Department of Animal Science, Michigan State University, East Lansing, MI 48824, USA; kimwons2@msu.edu; 5 Department of Animal Science and Technology, Sanghuh College of Life Sciences, Konkuk University, 120 Neungdong-ro, Gwangjin-gu, Seoul 05029, Korea; hglee66@konkuk.ac.kr

**Keywords:** mammary gland, epithelial barrier, mastitis, chemerin

## Abstract

**Simple Summary:**

This study evaluated the production and the function of chemerin, known as a chemoattractant and an antimicrobial protein, with regard to mammary epithelial defense in cows. The result demonstrated that mammary epithelial cells express chemerin protein. Chemerin treatment promoted the proliferation of bovine mammary epithelial cells and protected epithelial cells from oxidative stress. Meanwhile, mammary chemerin production was elevated by mastitis, which was possibly attributed to inflammatory cytokines. Therefore, the present study demonstrates the supportive ability of chemerin for mammary epithelial tissue and its regulation by inflammatory stimuli, suggesting its role in mammary epithelial defense against pathogen infection in cows.

**Abstract:**

Epithelial barrier function in the mammary gland acts as a forefront of the defense mechanism against mastitis, which is widespread and a major disorder in dairy production. Chemerin is a chemoattractant protein with potent antimicrobial ability, but its role in the mammary gland remains unelucidated. The aim of this study was to determine the function of chemerin in mammary epithelial tissue of dairy cows in lactation or dry-off periods. Mammary epithelial cells produced chemerin protein, and secreted chemerin was detected in milk samples. Chemerin treatment promoted the proliferation of cultured bovine mammary epithelial cells and protected the integrity of the epithelial cell layer from hydrogen peroxide (H_2_O_2_)-induced damage. Meanwhile, chemerin levels were higher in mammary tissue with mastitis. Tumor necrosis factor alpha (TNF-α) strongly upregulated the expression of the chemerin-coding gene (*RARRES2*) in mammary epithelial cells. Therefore, chemerin was suggested to support mammary epithelial cell growth and epithelial barrier function and to be regulated by inflammatory stimuli. Our results may indicate chemerin as a novel therapeutic target for diseases in the bovine mammary gland.

## 1. Introduction

Mastitis remains a widely spread disease in dairy production. Current prevention and cure methods for mastitis are mainly antibiotic-based. The administration of antibiotics might cause the emergence of drug-resistant pathogens. Thus, alternative solutions for mastitis have long been necessary [1]. The epithelial barrier system has an important immunological function, which prevents commensal and pathogenic microorganism infection. Particularly, epithelial innate immunity serves as the first line of defense against pathogens and has the potential to act coordinately with acquired immunity.

Antimicrobial peptides/proteins (AMPs) are one of the potent players of epithelial innate immunity, having a lower potential to cause antibiotic-resistant pathogens [2]. Therefore, AMPs in bovine mammary glands are thought to be a good target for the development of mastitis prevention and treatment methods. The type of AMPs differ among species; e.g., bovines do not possess α-defensin, a potent mucosal AMP in humans and mice [3]. This might imply that cows have a unique AMP system when compared with humans and rodents. Thus, revealing potent AMPs in the bovine mammary gland is necessary.

Chemerin, which was originally identified as a chemoattractant protein for antigen-presenting cells [4], is currently recognized to have antimicrobial properties [5]. This protein has an inhibitory effect on commensal pathogen activity, including *Escherichia coli* and *Staphylococcus aureus* which are the major mastitis-causing pathogens [6,7]. Taken together, chemerin has the potential to be a pivotal player of immunity against epithelial infection and thereby maintain tissue homeostasis. As we previously reported that *retinoic acid receptor responder 2* (*RARRES2*), encoding chemerin was highly expressed in cows’ mammary glands [8], we hypothesized that chemerin might act to regulate the mammary epithelial barrier mechanism. Studies including ours demonstrated that inflammatory cytokines strongly induce the expression of chemerin, which implies that immune response during mastitis pathogenesis or involution process might regulate chemerin signaling in the bovine mammary gland [9,10]. Therefore, the objectives of the present study were to identify chemerin-producing cells in mammary glands, and investigate the regulatory factor of its production, to understand its role in the mammary epithelial defense system.

## 2. Materials and Methods

### 2.1. Animals and Sample Collection

For gene expression evaluation of chemerin and its receptors, mammary gland and fresh milk samples were collected from multipara Holstein dairy cows in the lactating or dry-off period (*n* = 5 in total). For investigation of gene expressional change of chemerin, mammary gland and liver tissue were immediately separated from euthanized cows at 1 week (i.e., during the early-lactation stage; *n* = 3), 5 months (i.e., at the mid-lactation stage; *n* = 4), 9 months (i.e., at the late lactation stage; *n* = 3), and 15 months (i.e., at the dry-off period; *n* = 3) post parturition. The cows used in this experiment were multiparous (experiencing 3.1 ± 1.4 calving), except for two that were primiparous at the mid-lactation stage. All cows at late lactation were pregnant, whereas others were not. Euthanasia was performed by exsanguination from the carotid artery after a captive bolt or an overdose administration of sodium pentobarbital (Kyoritsu Seiyaku, Tokyo, Japan) via the jugular vein. Thereafter, all tissue samples were collected and frozen immediately in liquid nitrogen and stored at −80 °C for PCR and immunoblotting analyses or were fixed in phosphate-buffered 10% formalin for immunostaining. The other two multiparous Holstein dairy cows, in the dry-off period, were employed for the isolation and culture of peripheral blood mononuclear cells (PBMCs). Whole blood samples (20 mL) were drawn via jugular vein puncture using sodium heparin (10 IU/mL) (BD Biosciences, Billerica, MA, USA) as an anticoagulant. Blood samples were stored at room temperature and transferred to the laboratory for separation of PBMC.

### 2.2. Culture of Bovine Mammary Epithelial Cells

Immortalized bovine mammary epithelial cells (MAC-T cells) were cultured according to a previously described protocol [8]. Briefly, subculturing was performed in Low-Glucose Dulbecco’s Modified Eagle Medium (DMEM) (Wako Pure Chem, Osaka, Japan) supplemented with 10% fetal bovine serum (FBS) (GE Healthcare, Little Chalfont, UK), 1% penicillin-streptomycin-amphotericin B solution (Wako Pure Chem), 5 µg/mL bovine insulin (Sigma-Aldrich, St. Louis, MO, USA), and 1 µg/mL progesterone (Sigma-Aldrich) on a 100 mm plastic culture dish (Corning, Corning, NY, USA). MAC-T cells were induced for differentiation prior to cytokine treatment. Confluent MAC-T cells cultured in 12-well plates (Corning) were differentiated for 5 d in DMEM supplemented with 10% FBS, 1% penicillin-streptomycin-amphotericin B solution, 5 µg/mL bovine insulin, 10 µg/mL dexamethasone (Sigma-Aldrich), 5 µg/mL ovine prolactin (Sigma-Aldrich), and human growth hormone (Wako Pure Chem). Cells were cultured at 37 °C in a humidified CO_2_ incubator (5% CO_2_).

### 2.3. Cell Proliferation Assay

The proliferation of MAC-T cells was assessed by the 3-(4, 5-dimethylthiazol-2-yl)-2, 5-diphenyltetrazolium bromide (MTT) assay. MAC-T cells were seeded at 1000 cells/well in 96-well culture plates, and cultured in DMEM supplemented with 10% FBS and a 1% penicillin-streptomycin-amphotericin B suspension (Wako Pure Chem) for 10 h at 37 °C in a humidified CO_2_ incubator (5% CO_2_). Thereafter, the medium was removed and MAC-T cells were incubated with DMEM supplemented with 10% FBS, a 1% antibiotic mixture, and recombinant human chemerin (R&D Systems, Minneapolis, MN, USA) at 10, 100, or 300 ng/mL for 24 h at 37 °C, under 5% CO_2_. Following incubation, the MTT solution (5 mg/mL in 1× PBS) was added to each cell, followed by another 4 h incubation at 37 °C, 5% CO_2_. The supernatant was removed and 100 µL/well of 0.04 N HCl/isopropanol was added to dissolve the MTT formazan precipitate. An optical density (OD) measurement was performed at 570 nm with a DTX-880 microplate reader (Beckman Coulter, Brea, CA, USA).

### 2.4. Measurement of Extracellular Lactate Dehydrogenase (LDH) Level

MAC-T cells were cultured using the same procedure for the MTT assay and treated with chemerin at 10, 100, or 300 ng/mL for 24 h. Conditioned medium (50 μL) from each well was transferred to a new 96-well plate. Assay buffer was prepared as follows: mixing buffer A (4 mM iodonitrotetrazolium chloride in 0.2 M Tris-HCl, pH8.2), buffer B (6.4 mM nicotinamide adenine dinucleo-tide sodium salt, 320 mM lithium lactate, in 0.2 M Tris-HCl buffer), and buffer C (150 mM 1-methoxyphenazinemethosulfate in Tris buffer) at a ratio of 5000:5000:1. Each sample of conditioned medium was mixed with 50 μL of assay buffer and incubated for 1 h at 25 °C. Fifty microliters of 1 molar acetic acid was added to each well and the OD measurement was performed at 490 nm.

### 2.5. Measurement of Transepithelial Electrical Resistance (TEER)

The effect of chemerin on the integrity of MAC-T cells was investigated by monitoring the TEER across cell monolayers. MAC-T cells were seeded on cell culture inserts (Corning) and cultured until the required confluency was reached. Thereafter, the medium in the culture insert was replaced with a fresh subculturing medium containing 0.6 M H_2_O_2_ with/without recombinant human chemerin (10 or 100 ng/mL; R&D Systems) to incubate for 3 h. Cell culturing and incubation were conducted at 37 °C, 5% CO_2_, and 95% relative humidity. After incubation with H_2_O_2_ with/without chemerin, cell culturing plates with inserts were taken outside the CO_2_ incubator and a Millicell Electrical Resistance system (Millipore, Burlington, MA, USA) connected to electrodes was used to measure TEER values. Control experiments were also conducted under the same experimental conditions.

### 2.6. Isolation of Peripheral Blood Mononuclear Cells (PBMCs) and Measurement of Cell Proliferation

Isolation and cell proliferation assay of PBMCs were performed as previously described [11]. Briefly, whole blood samples were processed within 6 h of sample collection. PBMCs were separated by density gradient centrifugation; whole blood was diluted with 1× PBS and layered gently over Histopaque-1077 (Sigma-Aldrich). Isolated PBMCs were washed twice with 1× PBS. The PBMCs obtained were diluted with serum-free RPMI-1640 medium (Sigma-Aldrich) and the cell numbers were determined. The viability of PBMCs typically exceeded 85%, determined by trypan blue staining. The PBMCs were resuspended at 1 × 10^6^ viable cells/mL in Roswell Park Memorial Institute (RPMI) 1640 medium containing 25 mM 4-(2-hydroxyethyl)-1-piperazineethanesulfonic acid (HEPES) supplemented with heat-inactivated FBS, 2 mM L-glutamine, 100 U/mL of penicillin, 100 μg/mL of streptomycin, and 0.25 μg/mL of amphotericin B (all supplements were from Sigma-Aldrich) in 96-well plates. The PBMCs were incubated at 37 °C under 5% CO_2_ for 24 and 48 h with or without chemerin (100 ng/mL). Thereafter, cell proliferation was analyzed by the Cell Counting Kit-8 (CCK-8; Dojindo Molecular Technology, Kumamoto, Japan) according to the manufacturer’s protocol.

### 2.7. Cytokine Treatment of Bovine Mammary Epithelial Cells

Confluent MAC-T cells were washed twice with prewarmed 1× PBS and incubated in DMEM containing 10% FBS and a 1% penicillin-streptomycin-amphotericin B suspension supplemented with recombinant mouse tumor necrosis factor alpha (TNF-α) (1, 10 or 100 ng/mL; R&D Systems), recombinant human interleukin beta 1 (IL-β1) (0.5, 5, or 50 ng/mL; R&D Systems), or recombinant human INF-γ (1, 10, or 100 ng/mL; R&D Systems) for 24 h in a CO_2_ incubator. Treated cells were collected for gene expression analysis.

### 2.8. Gene Expression Analysis

Total RNA was extracted using RNAiso Plus (Takara Bio, Shiga, Japan). The purity and integrity of the extracted RNA were checked using a NanoDrop 1000 spectrophotometer (Thermo Fisher Scientific, Waltham, MA, USA) and agarose gel electrophoresis, respectively. First-strand cDNA was synthesized from 500 ng of total RNA using the PrimeScript RT Reagent Kit with the gDNA Eraser Perfect Real Time (Takara Bio), while no-input RNA reaction served as a negative control. RT-PCR for gene expression was performed using GoTaq Green Master Mix (Promega, Madison, WI, USA), followed by agarose gel electrophoresis stained by ethidium bromide. Quantitative RT-PCR (qRT-PCR), to investigate gene expressional levels of *RARRES2*, chemerin receptor genes (*chemokine-like receptor 1; CMKLR1, C-C motif chemokine receptor like 2; CCRL2, G protein-coupled receptor 1; GPR1*), and other genes related to mammary gland function, was performed using SYBR Premix Ex Taq II Tli RNaseH Plus (Takara Bio) with the Thermal Cycler Dice Real Time System II (Takara Bio). Reactions with no-input RNA or without cDNA served as negative controls. The primer sequences are shown in Table S1. The PCR efficiencies were checked by constructing standard curves using 5 points serial dilution of pooled cDNA. The reference genes for expressional level analysis were chosen from 18S rRNA, cyclin G-associated kinase (*GAK*), actin-beta (*ACTB*), or vacuolar protein sorting 4 homolog A (*VPS4A*) for each experiment based on their stability in the value of threshold cycle (Ct) among animal groups. Gene expression levels were calculated using the 2^−*dd*Ct^ method and described as relative values to the control group.

### 2.9. Immunoblotting

Plasma samples were diluted 10 times with PBS. Frozen mammary tissue samples or cultured MAC-T cells were homogenized in radioimmunoprecipitation assay buffer supplemented with 1 mM Na_3_VO_4_ and a protease inhibitor cocktail (Nacalai Tesque, Kyoto, Japan). Fresh milk samples were centrifuged at 18,000× *g* for 30 min and the intermediate clear phase was transferred to a new 1.5 mL tube. This was repeated once more to obtain soluble protein from fresh milk. To analyze chemerin abundance in mammary glands with and without mastitis, the soluble protein was extracted from paraffin-embedded tissue sections according to a published protocol [12]. A total of 10 µg of these protein samples or 10 µL of diluted plasma were separated by 15% SDS-PAGE and electroblotted onto a PVDF membrane. The membranes were blocked using 3% bovine serum albumin (BSA) in Tris-buffered saline and Tween 20 (TBST) for 30 min. Membranes were incubated with primary antibodies overnight at 4 °C, followed by incubation with secondary antibodies for 1 h at 25 °C. The antibodies used for Western blotting were as follows: anti-bovine chemerin (custom-made rabbit polyclonal IgG raised against antigen peptide; CVTSVDNAADTLFPAGQF, purified by antigen affinity chromatography), anti-β-actin (sc47778; Santa Cruz Biotechnology, Dallas, TX, USA), anti-rabbit IgG horseradish peroxidase (HRP)-conjugated (W4011; Promega), and anti-mouse IgG HRP-conjugated (W4021; Promega). Detection of the probed band was achieved using an enhanced chemiluminescence (ECL) Prime Western Blotting Detection System and ImageQuant LAS 4000 (GE Healthcare).

### 2.10. Immunohistochemistry (IHC)

Frozen mammary gland tissues from Holstein dairy cows were sectioned to a 10 μm thickness, air-dried for 30 min at 25 °C, and fixed with 10% formalin for 20 min. Alternatively, formalin-fixed paraffin-embedded (FFPE) mammary glands were sectioned, deparaffinized with xylene, and rehydrated with a graded series of ethanol. The FFPE blocks of normal and mastitic tissue were adapted from a previous study [13] using different animals from ones used for gene expression analysis or frozen sections mentioned in Section 2.1. The mammary gland tissues for FFPE were obtained from cows with healthy or naturally occurring mastitic udder, euthanized in a local slaughterhouse. FFPE tissue sections were subjected to antigen retrieval by autoclave treatment in citrate buffer (pH 6.0), followed by incubation in 1% peroxide diluted in methanol to inactivate endogenous peroxidase. Thereafter, frozen or FFPE tissue sections were blocked with blocking buffer (2% normal goat serum diluted in TBST). Sections were incubated with anti-bovine chemerin rabbit IgG (custom-made polyclonal antibody: described above), anti-human CMKLR1 rabbit IgG (ab82773; Abcam, Cambridge, UK), or anti-human CCRL2 rabbit IgG (LS-A1094; LifeSpan Biosciences, Seattle, WA, USA) diluted in Can Get Signal Immunostain (Toyobo, Osaka, Japan) at 4 °C overnight. Following washing in TBST, tissue sections were probed with diluted Goat Anti-Rabbit IgG H&L conjugated with Alexa Fluor 488 or HRP at 25 °C for 1 h. The HRP-labeled immunocomplex was visualized using the diaminobenzidine substrate. Sections were observed by fluorescent or bright-field microscopy. To evaluate the specificity of the custom-made chemerin antibody, polyclonal IgG was adsorbed by an antigen-immobilized sepharose column, and the column flow-through was used as a negative control.

### 2.11. Statistical Analysis

Numerical data from experiments are presented as means ± standard error (SE). Data were statistically analyzed using student’s t-test or one-way ANOVA followed by Tukey-Kramer test, depending on the number of experimental groups. Significant differences were declared when *p* < 0.05, and tendencies were declared when *p* < 0.10. All statistical analyses were performed using the statistical package R (v3.4.4; available as a free download from https://www.r-project.org, accessed on 8 November 2021).

## 3. Results

### 3.1. Expression of Chemerin in Bovine Mammary Gland

**Figure 1:**
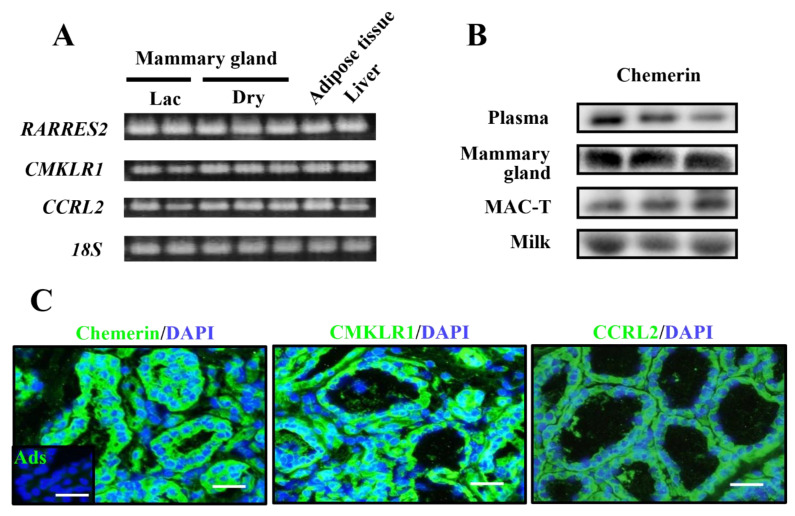
Expression and localization of chemerin and its receptors in the bovine mammary gland. Mammary gland tissues were collected from multiparous Holstein dairy cows at the lactation (Lac) and dry-off periods (Dry); *n* = 2 and 3, respectively. Gene expression of chemerin and its receptors were investigated by RT-PCR, whereas perirenal adipose tissue and the liver served as positive controls (**A**). Chemerin protein (approximately 18 kDa) was detected by immunoblotting in plasma and fresh milk from other lactating Holstein cows (*n* = 3) (**B**). Chemerin or its receptor-expressing cells were visualized by immunostaining in frozen mammary gland sections from lactating Holstein cows; *n* = 3 (**C**). Control sections were treated with antigen-adsorbed anti-chemerin antibodies for immunostaining (Ads). Counterstaining was performed with 4’,6-diamidino-2-phenylindole (DAPI). Scale bar = 50 μm.

*RARRES2* mRNA expression was detected in mammary glands from the lactation and dry-off period with a comparable level to white adipose tissue and the liver, which served as positive controls, according to our previous report [14] (Figure 1A). 

The expression of *CMKLR1* and *CCRL2* were also detected in mammary glands in the lactation and dry-off period. The mRNA of *GPR1* was observed in the dry-off period, with much lower mRNA abundance in the lactation period. Regarding protein expression, chemerin protein was detected in mammary tissue and immortalized bovine mammary epithelial cells, namely MAC-T cells (Figure 1B; whole blot images are shown in Figure S1). Importantly, extracellular chemerin was detected in fresh milk as well as in plasma. Histochemistry detected chemerin protein mainly in epithelial cells and in the stromal fraction with a weaker signal (Figure 1C). CMKLR1 and CCRL2 were detected in the epithelial area (Figure 1C). GPR1 was not examined, as its function in the immune system is still unknown. No signal was detected in histochemistry using antigen-adsorbed anti-chemerin IgG, ensuring its specificity. Antibodies’ reactivities were also assessed by staining the liver and adipose tissue sections (Figure S2).

### 3.2. Effect of Chemerin on Mammary Epithelial Cell Proliferation, Integrity, and Proliferation of PBMCs

**Figure 2:**
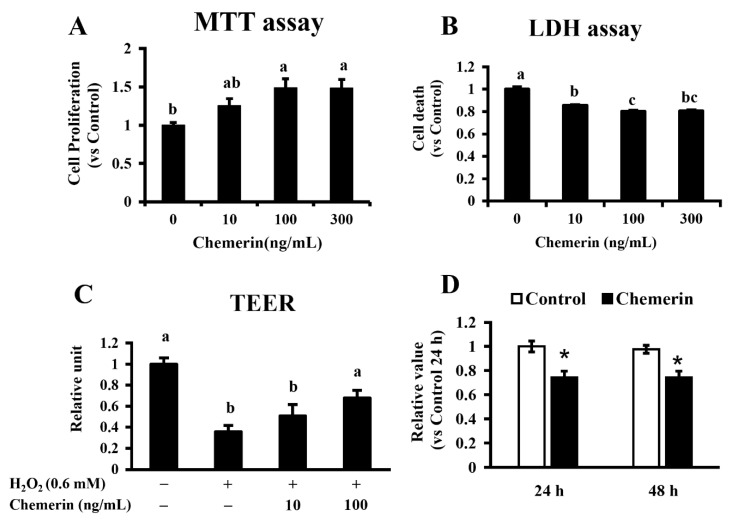
Protective effects of chemerin on mammary epithelial cells and PBMC. Immortalized bovine mammary epithelial cells (MAC-T cells) were cultivated in the presence of recombinant bovine chemerin for 24 h. Cellular proliferation and viability were measured by MTT (**A**) and LDH (**B**) assays. After reaching 100% confluency, MAC-T cells were cultured for 3 h in the presence/absence of H_2_O_2_ with/without bovine chemerin at the indicated concentrations. The transepithelial electrical resistance (TEER) assay was also performed for epithelial barrier function (**C**). The PBMCs were incubated for 24 and 48 h with chemerin (100 ng/mL), and cell proliferation was analyzed by the Cell Counting Kit-8 (CCK-8) (**D**). Different alphabet letters represent statistical significance: *p* < 0.05 by Tukey-Kramer test (**A**–**C**). Asterisks indicate *p* < 0.01 vs. the control by student’s *t*-test.

As our previous study reported that chemerin induced extracellular signal-regulated kinase (ERK) activity in MAC-T cells [8], we evaluated whether chemerin has a growth-promoting effect using the same cell line. Data from the MTT assay showed that the 24 h treatment of MAC-T cells with chemerin resulted in higher proliferation values (49% increases in 100 and 300 ng/mL treatments; *p* < 0.05) than that of the control (Figure 2A). In the LDH assay, MAC-T cells treated with chemerin showed a lower extracellular LDH level compared to that of the control (14% to 19% decreases; *p* < 0.05 for all) (Figure 2B). TEER was also measured to evaluate the protective effect of chemerin on epithelial integrity. Chemerin treatment on confluent MAC-T cells ameliorated the decreased TEER induced by H_2_O_2_ treatment (Figure 2C; 90% increases in 100 ng/mL treatment vs. H_2_O_2_ treatment; *p* < 0.05). Chemerin treatment inhibited the PBMCs proliferation (Figure 2D; 26% decreases at 24 and 48 h; *p* < 0.05).

### 3.3. Increased Chemerin Production in Mammary Gland with Mastitis

**Figure 3:**
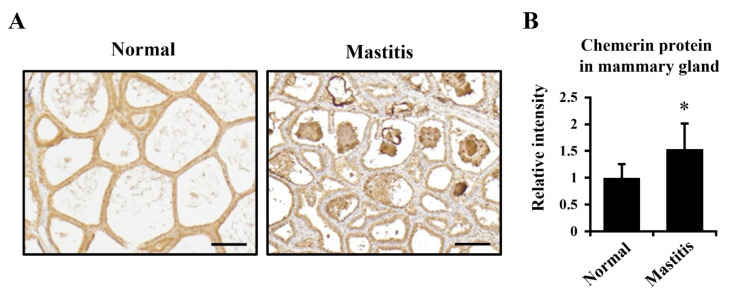
Elevated chemerin production in the mastitic mammary gland. (**A**) Paraffin-embedded sections of normal and mastitic mammary gland tissue from Holstein dairy cows were immunostained to visualize chemerin and its receptors. Scale bar = 200 μm. (**B**) Total protein was extracted from the sections and subjected to immunoblotting to compare relative levels of chemerin protein between normal and mastitic cows. * *p* < 0.05 by paired *t*-test.

The immunostaining of mammary glands from healthy and mastitis udders indicated that chemerin was produced in both conditions whereas chemerin was abundantly found in alveoli and ducts in the mastitic udder (Figure 3A). 

To compare the amount of chemerin in both conditions, total protein was extracted from the FFPE mammary glands, and subjected to immunoblotting (Figure 3B). The results showed relative chemerin abundance was higher in mastitic mammary glands (54% increase; *p* < 0.05).

### 3.4. Elevated Expression of Chemerin in Dairy Cows during the Dry-Off Period

**Figure 4:**
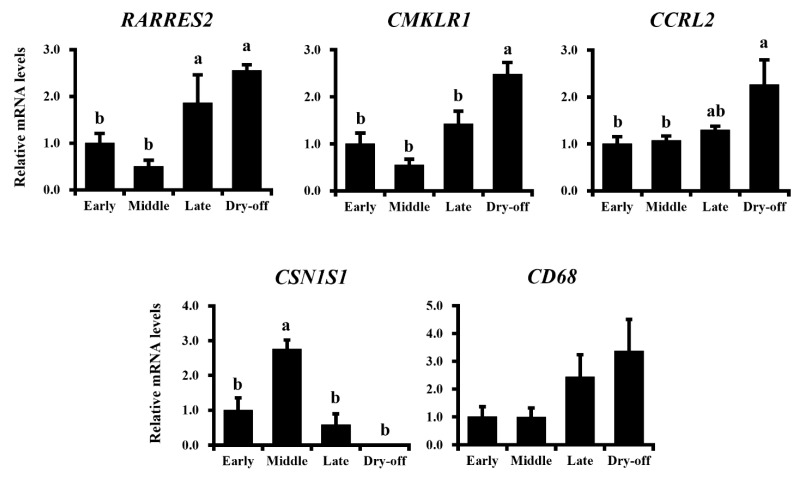
Expressional changes in chemerin, its receptors are associated with lactation periods in dairy cows. Mammary gland samples were collected from Holstein dairy cows at 1 week, 5 months, 9 months, and 15 months postpartum (described as Early, Middle, Late, and Dry-off; *n* = 3, 4, 3, and 3, respectively). Expressional levels of genes of *RARRES2* encoding chemerin and other genes of interest were investigated by qRT-PCR. Different letters represent statistical significance: *p* < 0.05 by Tukey-Kramer test.

To further investigate the relationship between chemerin production and immune response in mammary glands, the expression of chemerin was examined in the mammary glands of lactating or dry-off dairy cows (Figure 4). 

Lactation stages were confirmed by the expression of *CSN1S1* encoding alpha-casein, as the highest expression was observed in the mid-lactation period (175% increase vs. early-lactation; *p* < 0.05). The expression of *RARRES2* was higher in both late-lactation and dry-off periods (86% and 155% increases vs. early-lactation, respectively; *p* < 0.05 for both). The expression of *CMKLR1* and *CCRL2* were also higher in the dry-off period (148% and 126% increases vs. early-lactation, respectively; *p* < 0.05 for both). No difference in *CD68* expression was observed among lactation periods, as the surface marker of antigen-presenting cells which are the targets of chemerin (*p* > 0.1). However, its expression was numerically higher in the dry-off period.

### 3.5. Inflammatory Cytokines-Induced Upregulation of Chemerin and Its Receptor Expression in Mammary Epithelial Cells

**Figure 5:**
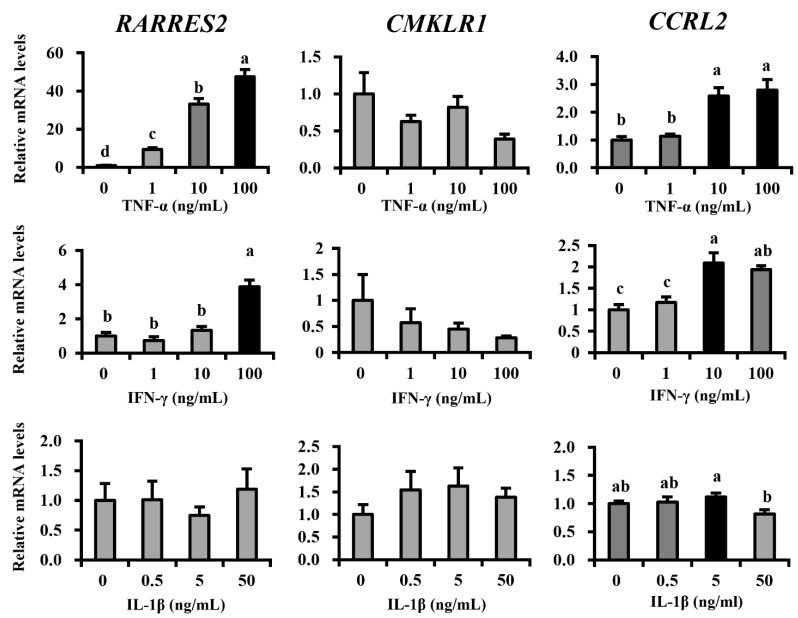
Inflammatory cytokines regulate chemerin and its receptor’s expression. Immortalized bovine mammary epithelial cells (MAC-T cells) were cultivated and differentiation was induced in vitro for 5 days. Differentiated MAC-T cells were treated with TNF-α, IL-1β, or IFN-γ for 24 h at indicated concentrations (*n* = 6 for each treatment concentration). Gene expression of RARRES2 encoding chemerin and genes encoding its receptors were investigated by qRT-PCR after treatments. Different letters represent statistical significance: *p* < 0.05 by Tukey’s HSD test.

To determine the regulatory factor of chemerin expression in mammary cells, we investigated the effect of mastitis-related proinflammatory cytokines on the gene expression of chemerin (Figure 5). The treatment of TNF-α on MAC-T cells dramatically upregulated gene expression of *RARRES2* in a dose-dependent manner (838%, 3223%, and 4642% increases in 1, 10, and 100 ng/mL treatments vs. control, respectively; *p* < 0.05 for all). *CCRL2* expression was also upregulated by TNF-α treatment (159% and 180% increases in 10 and 100 ng/mL treatments, respectively; *p* < 0.05 for both). In contrast, *CMKLR1* expression did not change after TNF-α treatment (*p* > 0.1). INF-γ also elevated the gene expression of chemerin at higher treatment concentrations (287% increase in 100 ng/mL; *p* < 0.05) as well as inducing *CCRL2* expression (109% and 94% increases in 10 and 100 ng/mL treatments, respectively; *p* < 0.05 for both), but not that of *CMKLR1* (*p* > 0.1). Alternatively, IL-1β did not affect *RARRES2* and its receptor’s expression, when compared to control (*p* > 0.1), except for a decrease in *CCRL2* at 50 ng/mL.

## 4. Discussion

The present study examined the potential role of chemerin in the bovine mammary gland, and also assessed its involvement in mastitis, considering its role as a chemokine [4,15], and its recently recognized antimicrobial potential [6]. The results of the expressional analysis demonstrated gene and protein expression of chemerin in mammary glands of dairy cows and its milk secretion. Chemerin was originally reported to be abundantly present in the inflammatory fluid and to have chemotactic potential to dendritic cells, macrophages, and natural killer (NK) cells, whereas its effect on monocytes is in deliberation [4]. Antigen-presenting cells, particularly macrophages, reside in healthy ma-mmary glands, which contribute to the monitoring of bacterial invasion via alveoli epithelial cells. The present results show basal production of chemerin in mammary epithelial cells, suggesting its contribution to the local innate immunity of mammary glands by recruiting these immune cells. Chemerin is an antimicrobial agent in the human epidermis and inhibits the growth of commensal pathogens including E. coli and S. aureus, which are known as major mastitis-causing pathogens. This property is attributed to the hydrophobic internal region of chemerin [6,7]. Another study reported the production of chemerin in intestinal epithelia of human fetuses and infants [16]. Thus, these reports indicate that chemerin produced in epithelial tissues has antimicrobial potential in addition to chemotactic ability, which is similar to complement factors or some specific types of chemokine [17]. Importantly, despite a lack of putative apical-sorting signal in its amino acid sequence, chemerin was secreted from both apical and basolateral sides in a study using an intestinal cell line [16]. This supports our data showing the detectable amount of secreted chemerin in milk. Thus, it is thought that chemerin potentially acts as an antimicrobial protein and a chemokine in a mammary epithelial barrier mechanism in dairy cows.

In addition to the reported functions of chemerin related to immunity, we previously revealed that chemerin upregulated GLUT1 and CSN3 (kappa-casein) expression in cultured mammary epithelial cells [8], suggesting its positive effect on mammary gland functions including lactation. As chemerin was shown to be produced in the cow’s mammary gland, this protein can act in a paracrine as well as endocrine manner. This led us to further investigate if chemerin has another regulatory effect on mammary epithelial cells. Already, some research exists indicating the proliferative effect of chemerin on some cell types, including vascular smooth muscle cells, and preadipocytes [18,19]. In the present study, the treatment of chemerin was shown to promote the proliferation and suppressed LDH release in MAC-T cells. Our previous study found that chemerin elevates intracellular ERK activity in MAC-T cells, supporting the present result. This suggests the promot-ive effect of chemerin on the growth and survival of mammary epithelial cells.

The mammary barrier function is largely attributed to epithelial integrity, mediated by tight junctions. In the case of mastitis, epithelial integrity is impaired by inflammatory stimuli of molecules such as bacterial lipopolysaccharide (LPS), inflammatory cytokines, and reactive oxygen species (ROS) [20]. We treated MAC-T cells with H_2_O_2_ to mimic ROS-induced damage in mastitis and evaluated the effect of chemerin in this process. The results showed that chemerin protected MAC-T cells from H_2_O_2_ induced epithelial disintegration. It is reported that elevated ROS and nuclear factor kappa-light-chain-enhancer of activated B cells (NF-κB) activity in mammary epithelial cells impairs cellular polarity, which is necessary for epithelial integrity [21]. A previous study showed that chemerin inhibits NF-κB activity induced by inflammatory cytokine stimuli [22]. This supports our result of the protective effect of chemerin on the epithelial barrier. The result that chemerin suppressed proliferation of PBMCs might simultaneously suppress the overactivity of immune response to maintain epithelial integrity. However, a previous report also indicated that chemerin activates the PI3K/Akt pathway, which might be involved in the dysfunction of epithelial integrity [18]. Thus, the mechanism of chemerin on epithelial integrity requires careful investigation in future studies. Therefore, our results demonstrate the potential protective effect of chemerin on bovine mammary glands by preventing impaired epithelial integrity.

The present study suggested the role of chemerin in the inflammatory response in mammary glands because its production was upregulated in mastitis. Whereas the recruitment of neutrophils takes place acutely during mastitis, the simultaneous infiltration of macrophages is thought to occur in mammary epithelia [23]. A previous study demonstrated that the stimulation of bovine mammary glands with LPS, lipoteichoic acid, and/or peptidoglycan induced upregulation of monocyte chemotactic protein-1 (MCP-1) production, a well-known chemokine for macrophages [24]. Thus, chemerin might be involved in this step with its chemotactic activity. Alternatively, it is also reported that proteolytic activation of chemerin is performed by *S. aureus*-derived serine proteases, as well as by cathepsins as host proteases, which are necessary for developing its chemotactic activity [7]. This suggests a synergistic activation of chemerin by host and bacterial proteases to lead to the acute inflammatory response in the infected/damaged region of mammary tissue.

Our data demonstrated higher expression of chemerin in involuted mammary glands of non-pregnant dry-off cows. Following the onset of involution, secreted matrix metalloproteases activate chemokines to recruit immune cells into mammary glands for the depletion of apoptotic mammary epithelial cells. During this process, macrophages are recruited to remove apoptotic cells, resulting in the occupation of stromal cells in tissue [25]. Additionally, Hu et al. reported that chemerin induced the activities of autophagy and apoptosis in cultured bovine mammary epithelial cells (BMECs) [26]. The chemerin-induced apoptosis was further activated when autophagy was inhibited by chloroquine. This implicates that chemerin can induce the programmed death of bovine mammary epithelial cells during the process of involution and maintenance of the mammary gland during the dry-off period [26]. Therefore, chemerin was suggested to act as a chemokine to antigen-presenting cells (APCs), and also as an inducer of apoptosis in involuting/involuted mammary glands, which supports the closed relationship between chemerin and the immune response in mammary glands. However, the difference in the direct effect of chemerin on bovine mammary epithelial cells observed in the study indicated above and the present study, promoting cell death or survival, might be dependent on lactation period and hormonal status, and must be investigated in detail in a future study.

Here, we further tried to determine the regulatory factors of mammary epithelial chemerin expression. During the process of mastitis pathogenesis, the production of pro-inflammatory cytokines is acutely induced to activate immune responses: firstly IL-8 attracts neutrophils, followed by TNF-α, interferon gamma (IFN-γ), and IL-1β induces tissue inflammation [27]. Our results showed that TNF-α strongly upregulated chemerin expression, as well as high-dose INF-γ. The effect of TNF-α on chemerin upregulation was consistent with the previous studies, including ours, using various cell types: epithelial cells, fibroblasts, and adipocytes [28]. Meanwhile, the response of chemerin receptor expression for these cytokines varied between each receptor. The expression of *CMKLR1*, a functional receptor for chemotaxis induction, was not affected by all cytokines used to treat MAC-T cells. Future studies should investigate its expression in APCs. *CCRL2* expression was upregulated by TNF-α and IFN-γ. CCRL2 was reported to capture chemerin on the cell surface for local concentration in supporting chemotaxis [29]. Thus, this upregulation was beneficial for APC recruitment in inflammatory conditions in mammary glands. It could be concluded that TNF-α, of the assessed cytokines, is the most potent upregulator of chemerin in mastitis, which contributes to APC recruitment to the inflammatory site, in combination with upregulated *CCRL2*.

## 5. Conclusions

This study revealed the production of chemerin and its regulatory cytokines in mammary epithelial cells and investigated its physiological role in mammary glands. The results showed that chemerin acts as a positive factor for mammary epithelial cell growth and barrier function. This protein was abundantly produced during mastitis, indicating its role in the defense system via its supportive effect on cell growth and integrity of epithelial cells, as well as its previously reported antibacterial and chemotactic abilities. These data suggest chemerin as an important endogenous factor against pathogens in the bovine mammary gland and as a potential therapeutic target for mastitis. Future studies should clarify the detailed regulatory mechanism of chemerin production and find natural compounds to modulate chemerin function for its application in dairy production.

## Data Availability

The data presented in this study are available in this article or as Supplementary Material.

## Supplementary Materials

The following are available online at https://www.mdpi.com/article/10.3390/ani11113194/s1, Table S1: Sequences of primers used for RT-PCR and quantitative RT-PCR (qRT-PCR); Figure S1: Whole blot images for the immunoblotting; Figure S2: Reactivities of anti-chemerin, anti-CMKLR1 and anti-CCRL2 antibodies.
