# Supplementary material for: Chemerin Regulates Epithelial Barrier Function of Mammary Glands in Dairy Cows
Source: Animals (Basel). 2021 Nov 9;11(11):3194. doi: 10.3390/ani11113194 (PMC8614423; doi:10.3390/ani11113194)
Supplement: Supplementary file 1 [file animals-11-03194-s001.zip › Table_S1.pdf]

**Table S1**. Sequences of primers used for RT-PCR and quantitative RT-PCR (qRT-PCR)

| Gene | Accession No | | Sequences (5' - 3') | Length (bp) | Efficiency[C] | ($R^2$)[D] |
|---|---|---|---|---|---|---|
| *RN18S1*[A] | NR_036642.1 | F | AGTACGCACGGCCGGTACAGT | 130 | - | - |
| | | R | CAGCGCCCGTCGGCATGTATT | | | |
| *GAK*[B] | NM_001046084.2 | F | TCTGGGAAGTGGCAGAGAGT | 294 | 1.85 | 0.99 |
| | | R | CGGCACGTCTGGTAGAAGAT | | | |
| *VPS4A*[B] | NM_001046615.1 | F | CAAAGCCAAGGAGAGCATTC | 222 | 1.92 | 0.97 |
| | | R | ATGTTGGGCTTCTCCATCAC | | | |
| *ACTB*[B] | NM_173979.3 | F | GAATCCTGCGGCATTCACGA | 192 | 1.94 | 0.99 |
| | | R | AGGGGGCGCGATGATCTTG | | | |
| *RARRES2*[B] | NM_001046020.2 | F | GTTTGTGAGGCTGGAGTTC | 173 | 1.95 | 0.99 |
| | | R | GAGTCTGTATGGGACAGTGC | | | |
| *CMKLR1*[B] | NM_001145235.1 | F | CATCGTCGTTCTGGAGGAGT | 169 | 2.00 | 0.97 |
| | | R | GTTGAGGAACCAGACGGTGT | | | |
| *CCRL2*[B] | NM_001075732.2 | F | CCAGGGGGATAAACAGTGGT | 266 | 2.04 | 0.98 |
| | | R | ATCTTGGGGTCGTATGGGGT | | | |
| *GPR1*[B] | NM_001206545.1 | F | GGTCTCCTTGGTGTTGTGCT | 291 | 1.97 | 0.98 |
| | | R | TAGCGGTCCAGGCTTATCAC | | | |
| *CD68* | NM_001045902.1 | F | AGGTTGGCTGTGTTCTTCTCT | 183 | 1.92 | 0.99 |
| | | R | GGTTCTGTGGCTCTTGGTAGT | | | |
| *CSN1S1* | NM_181029.2 | F | TGTCTTGTGGCTGTTGCTCT | 76 | 1.94 | 0.97 |
| | | R | CATTGAGGACTTCTTGAGGGA | | | |

A: 18S rRNA was not used for qRT-PCR. Thus, primer efficiency and $R^2$ were not determined.

B: These primer pairs were incorporated from Suzuki *et al*., 2016

C: Primer efficiency = $10^{(-1/Slope)}$; the slope was calculated from a standard curve constructed using a 5 points dilution series of pooled cDNA

B: $R^2$ represents the coefficient of determination for the standard curve.
