# Supplementary figures and images for: Chemerin Regulates Epithelial Barrier Function of Mammary Glands in Dairy Cows
Source: Animals (Basel). 2021 Nov 9;11(11):3194. doi: 10.3390/ani11113194 (PMC8614423; doi:10.3390/ani11113194)

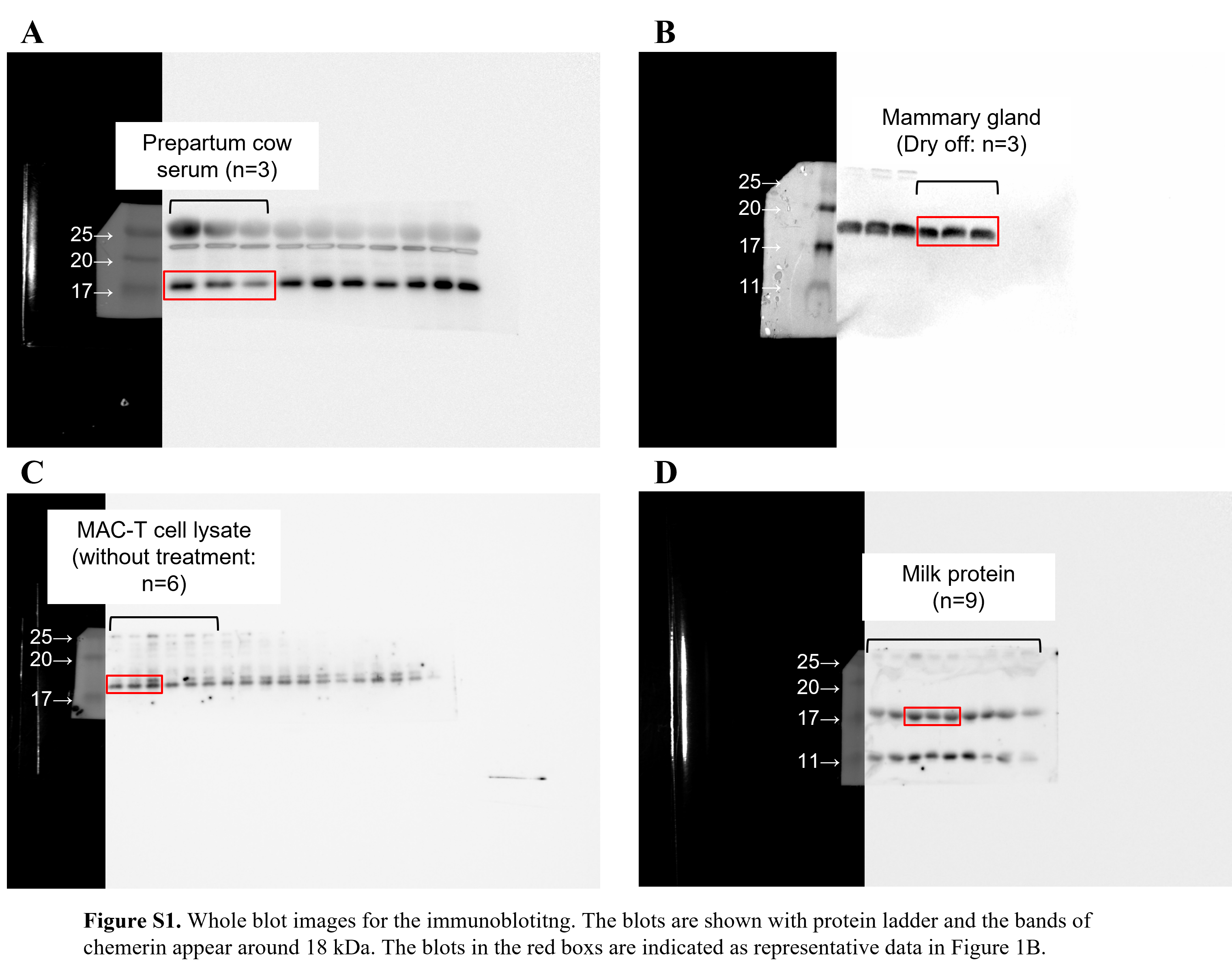

Supplement: Supplementary file 1 [file animals-11-03194-s001.zip › Figure_S1.tif]

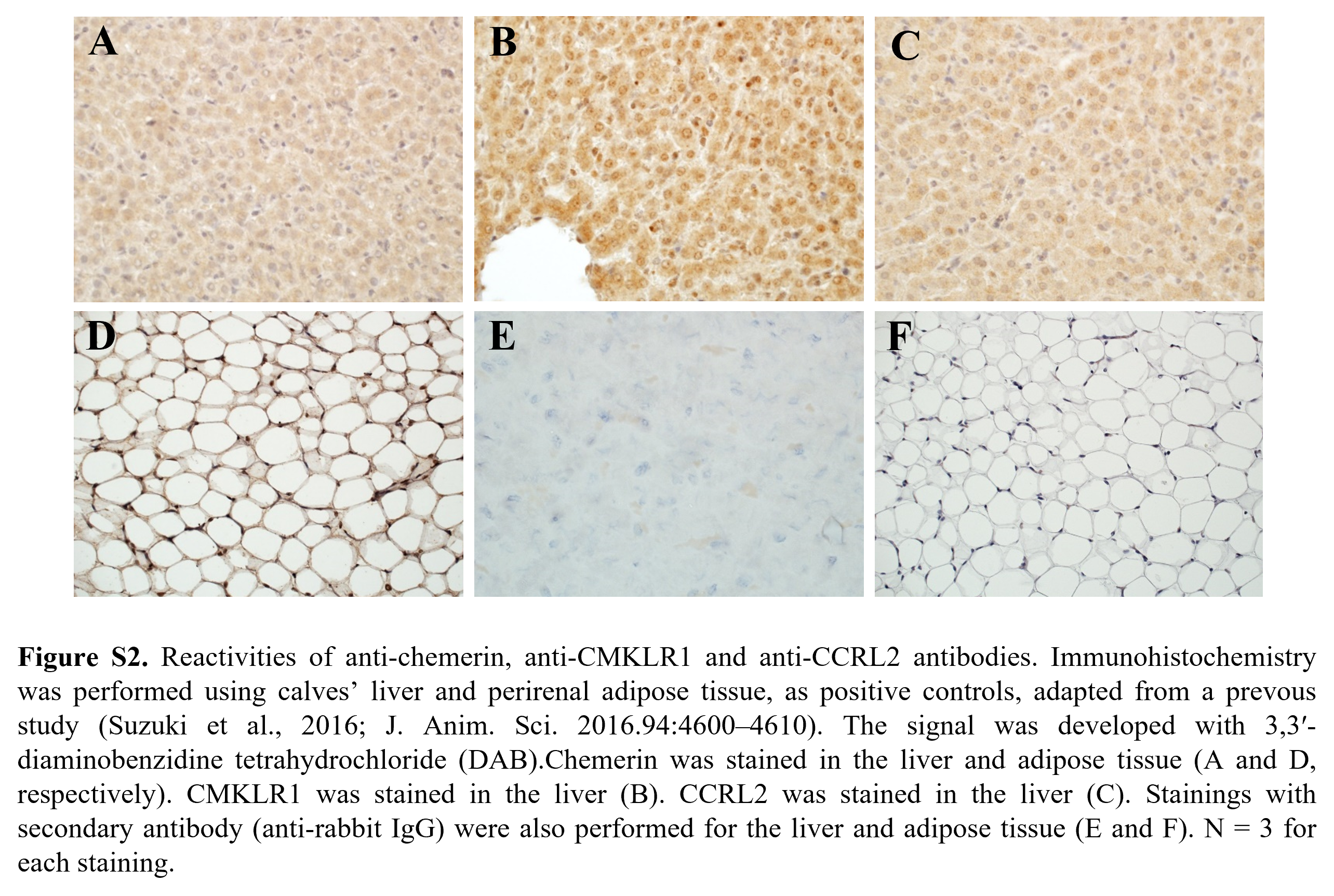

Supplement: Supplementary file 1 [file animals-11-03194-s001.zip › Figure_S2.tif]
